# Case Report: Subtherapeutic Vancomycin and Meropenem Concentrations due to Augmented Renal Clearance in a Patient With Intracranial Infection Caused by *Streptococcus intermedius*

**DOI:** 10.3389/fphar.2021.728075

**Published:** 2021-10-06

**Authors:** Marcus Fransson, Anders Helldén, Åse Östholm Balkhed, Dženeta Nezirević Dernroth, Maria Ha, Mats Haglund, Peter Milos, Håkan Hanberger, Bertil Kågedal

**Affiliations:** ^1^ Department of Neurosurgery and Department of Biomedical and Clinical Sciences, Linköping University, Linköping, Sweden; ^2^ Department of Clinical Chemistry and Clinical Pharmacology and Department of Biomedical and Clinical Sciences, Linköping University, Linköping, Sweden; ^3^ Department of Infectious Diseases, and Department of Biomedical and Clinical Sciences, Linköping University, Linköping, Sweden; ^4^ Department of Infectious Diseases, Kalmar County Hospital, Kalmar, Sweden

**Keywords:** suboptimal antibiotic treatment, tubular cell membrane transporters, glomerular filtration rate, augmented renal clearance, vancomycin clearance, iohexol clearance, creatinine clearance, urea clearance

## Abstract

*Streptococcus intermedius* occasionally causes brain abscesses that can be life-threatening, requiring prompt antibiotic and neurosurgical treatment. The source is often dental, and it may spread to the eye or the brain parenchyma. We report the case of a 34-year-old man with signs of apical periodontitis, endophthalmitis, and multiple brain abscesses caused by *Streptococcus intermedius*. Initial treatment with meropenem and vancomycin was unsuccessful due to subtherapeutic concentrations, despite recommended dosages. Adequate concentrations could be reached only after increasing the dose of meropenem to 16 g/day and vancomycin to 1.5 g × 4. The patient exhibited high creatinine clearance consistent with augmented renal clearance, although iohexol and cystatin C clearances were normal. Plasma free vancomycin clearance followed that of creatinine. A one-day dose of trimethoprim–sulfamethoxazole led to an increase in serum creatinine and a decrease in both creatinine and urea clearances. These results indicate that increased tubular secretion of the drugs was the cause of suboptimal antibiotic treatment. The patient eventually recovered, but his left eye needed enucleation. Our case illustrates that augmented renal clearance can jeopardize the treatment of serious bacterial infections and that high doses of antibiotics are needed to achieve therapeutic concentrations in such cases. The mechanisms for regulation of kidney tubular transporters of creatinine, urea, vancomycin, and meropenem in critically ill patients are discussed.

## Introduction

Augmented renal clearance (ARC), defined as a measured creatinine clearance >130 ml/min/1.73 m^2^, is increasingly being recognized in critically ill patients. The condition can lead to subtherapeutic concentrations of renally excreted drugs. The case-patient presented with low serum creatinine concentrations and high creatinine clearance, leading to the diagnosis of ARC. The pathophysiology of ARC is unknown, but high cardiac output, increased tubular creatinine secretion, and hormonal effects have been suggested. ARC may jeopardize the treatment of life-threatening deep bacterial infections with, for instance, members of the *Streptococcus anginosus* group. We report a patient with *S. intermedius* infection, endogenous endophthalmitis, and septic embolization to the CNS, arising from a dental infection. Increased drug elimination due to ARC resulted in subtherapeutic antibiotic drug concentrations until high doses were administered. The purpose of this case report is to illustrate the possible causes for rapid elimination of the drugs.

### Case Description

**FIGURE 1:**
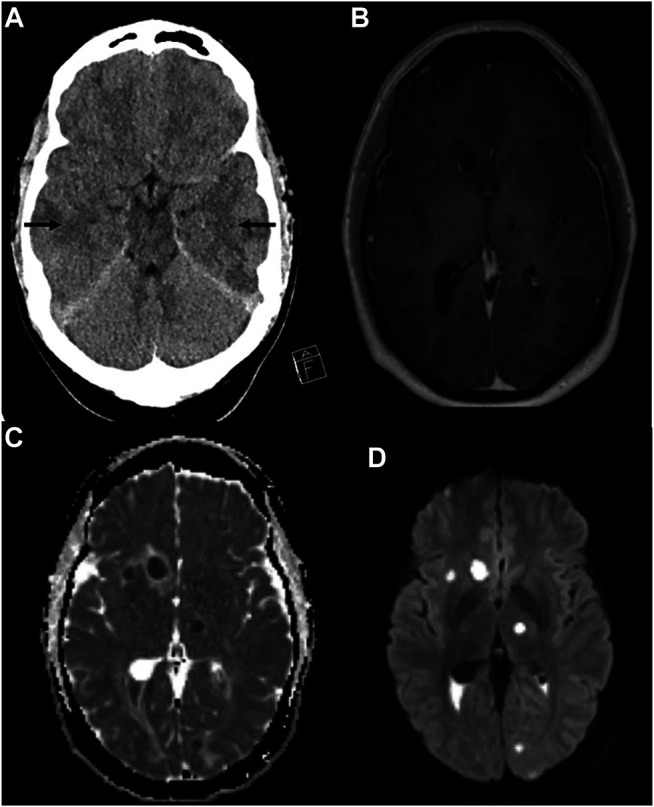
Computer tomography showing hypodense areas bilaterally in the temporal lobes indicating edema (arrows) **(A)**. MRI of the brain with T1WI with gadolinium contrast **(B)**, apparent diffusion coefficient (ADC) image **(C)**, and diffusion-weighted imaging (DWI) **(D)**. Multiple peripherally contrast-enhancing lesions with perilesional edema were found both supra- and infratentorial **(B)**. A thin ependymal contrast enhancement is found. Diffusion imaging **(C,D)** show reduced signaling in the parenchymal lesions and in the content of the posterior horns, indicating multiple abscesses and ventriculitis.

A 34-year-old man presented at the emergency room with 1-day history of headache, fever, photosensitivity, and visual impairment. On examination, he was awake but slightly disoriented (Glasgow Coma Scale 14). The following were observed: CRP: 50 mg/L, lumbar puncture: 25 cm H_2_O opening pressure (Lee and Lueck, 2014), CSF: white cell count of 7210 × 10^6^/L (6380 × 10^6^/L polymorphonuclear), lactate 8.2 mmol/L, and albumin 880 mg/L. Meningitis was diagnosed and treatment started with meropenem 2 g × 3, acyclovir 800 mg × 3, and betamethasone 8 mg × 4. CT brain showed no sign of abscess or empyema; bilateral diffuse low-density areas were observed in the frontal and temporal lobes, indicating edema (Figure 1A). Methods, reference values, and drug target values are given in Table 1.

**TABLE 1:** Definitions, methods, and reference values.

Clinical investigation and analysis	Reference intervals, target values	Methods and references
Clinical evaluation		
Glasgow Coma Scale (GCS)	3–15	Definition: The GCS is the summation of scores for eye, verbal, and motor responses Teasdale and Jennett (1974) and Teasdale and Jennett (1976). The minimum score is 3, which indicates deep coma or a brain dead state. The maximum is 15, which indicates a fully awake patient
Lumbar puncture opening pressure	6–25 cm H_2_O	Normal range: Whiteley et al. (2006) and Lee and Lueck (2014). The patient should be placed in lateral decubitus position
Quantiferon	Negative	Quantiferon is a test for presence of active or latent tuberculosis Sotgiu et al. (2019)
Cerebrospinal fluid, CSF		
CSF--albumin	<320 mg/L	Reference interval: personal information from neurochemistry laboratory, Mölndal
		Method: Immunoturbidimetric determination using the cobas c501/701 instruments. Calibrated against ERM-DA470k/IFCC reference material
CSF--lactate	<2,1 mmol/L	Reference interval: Lindgren et al. (2019)
		Method: Enzymatic determination using the cobas c501/701 instruments
CSF--WBC	0–5 × 10^6^/L	Reference interval: Traditional
		Method: Cell counts by manual microscopy or phase contrast microscopy using the Fuchs-Rosenthal or Bürker chambers
CSF vancomycin	6–19 mg/L	Target value: Albanèse et al. (2000) and Shokouhi and Alavi Darazam (2014)
		Method: The spectrophotometric homogenous enzyme immune technique used for serum was validated for use as the method for CSF in a correlation study with an HPLC method Marks et al. (2019)
Plasma or serum and urine		
P--C-reactive protein	<10 mg/L	Method: Particle enhanced Immunoturbidimetric determination using the instruments cobas c501/701. Calibrated against ERM-DA470k/IFCC reference material
P--Creatinine	61–108 µmol/L	Reference interval: Erlandsen and Randers (2018)
		Method: Enzyme method using cobas c 501/701 instruments from Roche Diagnostics
		Creatinine was calibrated using the calibrator for automated systems (C.f.a.s) traceable to the IDMS reference creatinine method
U--Creatinine	5–22 mmol/24 h	Reference values: Junge et al. (2004)
		Method: Enzyme method using cobas c 501/701 instruments from Roche Diagnostics. The method is standardized against the ID/MS calibrator for automated systems (C.f.a.s). The amount excreted per 24 h (mmol/24 h) is calculated as concentration (mmol/L) × volume (L/24 h)
P--Iohexol		Method: Iohexol analysis was performed on an Acquity UPLC I-Class UHPLC system coupled with a Xevo TQ-S micro Triple Quadrupole Mass Spectrometer (Waters). Iohexol (Nycomed Amersham AB) and d5-iohexol, the internal standard, (ALSACHIM SAS) were simultaneously eluted by a BEH C18 column (Waters) using a gradient of 0.1% formic acid in 2 mM ammonium acetate and methanol. Simultaneous peak integration and quantitation for iohexol were achieved automatically using MassLynx Software
P--Cystatin C	0.62–1.04 mg/L	Reference interval: Erlandsen and Randers (2018)
		Method: Particle-enhanced turbidimetric analysis according to principles given by Kyhse-Andersen et al. (1994). Calibrator: C.f.a.s. The method is calibrated against ERM-DA471/IFCCs reference material Grubb et al. (2014)
S--Vancomycin, total	15–20 mg/L	Target values: Rybak et al. (2009) and Rybak et al. (2020)
		Method: Spectrophotometric homogenous enzyme immune technique using cobas c 502 instrument from Roche Diagnostics
		Calibrator: Preciset TDM I calibrators
S--Vancomycin, free	10.5–14 mg/L	Target value: Can be estimated as 0.7 × mg/L Sokol (1991), Uchino et al. (2000), Fanos and Cataldi (2001), Shibayama et al. (2007), Klein et al. (2011), Pais et al. (2020)
		Method: For ultrafiltration of serum, Nanosep Omega PES (Millipore), with a 10 kDa cutoff was used. For chromatographic analysis, we used a Waters Alliance 2695 HPLC system equipped with a Waters bridge column BEH C18 (2.5 μm × 3 mm x 75 mm). Detection was with a photodiode array detector (Waters), and Chromeleon v.7 was used for data collection and analysis
P--Meropenem	>8 mg/L	Target value: “Total trough concentrations above 8.0 mg/L for meropenem and above 22.5 mg/L for piperacillin were defined as the breakpoints for target attainment” Scharf et al. (2020)
		Method: Total meropenem was analyzed at the Department of Clinical Pharmacology, Karolinska University Hospital, using an accredited LC-MS/MS method
Clearances		
Creatinine clearance, endogenous	66–143	Reference interval: Junge et al. (2004)
		Method: Urine collection for 12 h. Collection of urine was from midnight (00.00) until 12.00. Plasma creatinine concentrations used for clearance calculations were from early morning the same day
		Clearance was calculated according to the formula Cl_Crea_ (ml/min) = urinary concentration (mmol/L) × urinary volume (L)/serum concentration (mmol/L) × collection time (min). The clearance values were normalized to standard body surface area of 1.73 m^2^ calculated from height and weight according to Dubois and Dubois (1989)
Iohexol clearance (GFR_iohexol_)	78–122 ml/min/1.73 m^2^	Reference values: Bäck et al. (1989)
		Method: After injection and 4-points measurements iohexol plasma clearance was calculated according to Bröchner-Mortensen (1972). Clearance day 9 was calculated from the dose (67 ml) given at the Department of Radiology and clearance day 14 from the 5 ml dose used in our standard method Krutzén et al. (1984). The clearance values were normalized to standard body surface area of 1.73 m^2^ calculated from height and weight according to Dubois and Dubois (1989)
Urea clearance	9.6–60 ml/min/1.73 m^2^	Reference values: Koch et al. (1980)
		Method: Urine collection for 12 h. Collection of urine was from midnight (00.00) until 12.00. Plasma urea concentrations used for clearance calculations were from early morning the same day
		Clearance was calculated according to the formula Cl_Urea_ (ml/min) = urinary concentration (mmol/L) × urinary volume (L)/serum concentration (mmol/L) × collection time (min). The clearance values were normalized to standard body surface area of 1.73 m^2^ calculated from height and weight according to Dubois and Dubois
Vancomycin clearance from total serum concentrations	50–157 (*n* = 10) or <118 ml/min/1.73 m^2^ from intersection at creatinine clearance of 130 ml/min/1.73 m^2^	Reference intervals: Rodvold et al. (1988)
		Clearance of healthy subjects
		Method: Blood samples for vancomycin concentrations were collected shortly before the dose, immediately after the two hour infusion, and at 3, 4, and 6 hours after the start of the infusion, and as a trough sample before next dose. Vancomycin clearance calculated using the trapezoidal rule and area under the curve (AUC)
		The clearance values were normalized to standard body surface area of 1.73 m^2^ calculated from height and weight according to Dubois and Dubois (1989)
Vancomycin clearance from free serum concentrations	x–y mL/min	Reference intervals: Not available in the literature
	x–y mL/min/1.73 m^2^	Method: Vancomycin clearance from free concentrations was obtained and calculated according to the same methods as from total vancomycin in serum
		The clearance values were normalized to standard body surface area of 1.73 m^2^ calculated from height and weight according to Dubois and Dubois (1989)
Cystatin C clearance (eGFR_cystatin C_)	mL/min/1.73 m^2^	Reference interval: Ca 15% less than iohexol clearance, see Figure 1A in Grubb et al. (2014)
		Method: Grubb et al. (2014) estimated clearance from plasma cystatin C was calculated according to the equation eGFR = 130 × Cystatin C^−1.069^ × age^−0.117^ − 7

His visual impairment worsened, perceiving only light and dark with his left eye. On day 7, MRI with contrast enhancement revealed multiple brain abscesses (Figures 1B–D) and reduced diffusion, indicating ventriculitis. Reduced diffusion was also observed in the retina of the left eye, suggesting abscess and retinal detachment.

The patient was transferred to the university hospital’s neurosurgical intensive care unit. CSF cultures were negative for bacteria, fungi, and neurotropic viruses. The QuantiFERON test and the tests for HIV and toxoplasmosis were also negative. The echocardiography indicated no endocarditis or other abnormality. The MRI showed no signs of thrombosis in the jugular vein, indicative of Lemierre’s syndrome. The intravitreal and subconjunctival injections of vancomycin and ceftazidime, as well as ocular dexamethasone and cyclopentolate, were administered. A puncture of a subcortical abscess in the right frontal lobe and the placement of an external ventricular drainage were performed.

Day 13 dental examination showed apical periodontitis requiring extraction of one mandibular and three maxillary teeth was identified. Two days later, surgical extirpation of a right frontal cortical abscess was performed. No bacterial growth was seen in cultures, but the material from the brain abscess was sent for 16S rRNA sequencing analysis. *S. intermedius* was detected and confirmed by PCR analysis. The CSF profiles improved, and day 21 MRI showed slight regression of ventriculitis and some abscesses. Intrathecal antibiotics were discontinued, and the ventricular drainage was removed. Ophthalmological examination showed almost complete retinal detachment with rupture. The eye was beyond help and thus enucleated. The patient was discharged after 6 weeks. Fourteen weeks after admission, the patient still acknowledged cognitive and memory impairments; fatigue; changed sense of smell, sound sensitivity, and photosensitivity; recurrent nightmares; and impaired motor function.

### Antibiotic Treatment

Initial treatment was meropenem 2 g × 3 and acyclovir 800 mg × 3. This was changed on day 7 to cefotaxime and metronidazole following the finding of multiple intracerebral abscesses (Table 2). To broaden antibacterial coverage, this was reversed the following day to meropenem and metronidazole, with the addition of intravenous vancomycin, and from day 9, intrathecal vancomycin was administered. On suspicion of toxoplasmosis, he received trimethoprim–sulfamethoxazole (TMP-SMX) on day 11, but this was replaced by rifampicin on day 12.

**TABLE 2:** Antibiotic treatment and drug concentrations in a neurosurgery patient with augmented renal clearance and multiple abscesses in the brain due to *S. intermedius* infection.

Day (day 1 is first day with symptoms)	3	4	5	6	7	8	9	10	11	12	13	14	15	16	17	18	19	20	21
Meropenem dosage	2 g × 3	→	→	→		2 g × 4	→	→	→	→	→	3 g bolus +12 g/day^b^	12 g/day^b^	16 g/day^b^	→	→	12 g/day^b^	→	→
Meropenem trough concentration (mg/L) (> 8 mg/L)^a^										1.3			11		23		31	17	
Acyclovir	800 mg × 3	→	→	→															
Betamethasone	8 mg × 4	→	→	→															
Cefotaxime					3 g × 4														
Metronidazole					1.5 g	0.5 g × 3	→	→	→	→	→	→	→	→	→	→	→	→	→
Vancomycin dosage						2 g bolus + 1.5 g ×3	1.5 g × 3	2 g × 3	→	2 g × 4	2 g × 3	1.5 g × 4	→	→	→	→	→	→	→
Vancomycin trough concentration (mg/L) (15–20 mg)^a^							6.5	9.8	9.0	22.3	11.7		14.6/12.6	12.0		15.1	19.6	18.7	19.3
Trimethoprim–sulfamethoxazole (TMP-SMX) (16 mg/ml, 80 mg/ml)									40 ml × 2										
Trimethoprim trough concentration (mg/L)										2.8									
Sulfamethoxazole trough concentration (mg/L)										75.8									
Rifampicin										450 mg × 2	→	600 mg × 2	→						
Vancomycin intrathecal dosage (mg single dosage)							10		10	10		10		20	20		20		20
Vancomycin intrathecal (CSF) trough concentration (mg/L)								<4		<4		<4		<4	<4	11.9	<4	12.4	<4

a Reference values.

b Continuous infusion.

Despite doses ranging from 1.5 g × 3 to 2 g × 4, a stable vancomycin target level of 15–20 mg/L (Rybak et al., 2020) was not achieved until 1.5 g × 4 was given (Table 2). Serum and CSF antibiotic concentrations remained undetectable or subtherapeutic until doses higher than those recommended for meningitis were administered.

### Research Investigations

**FIGURE 2:**
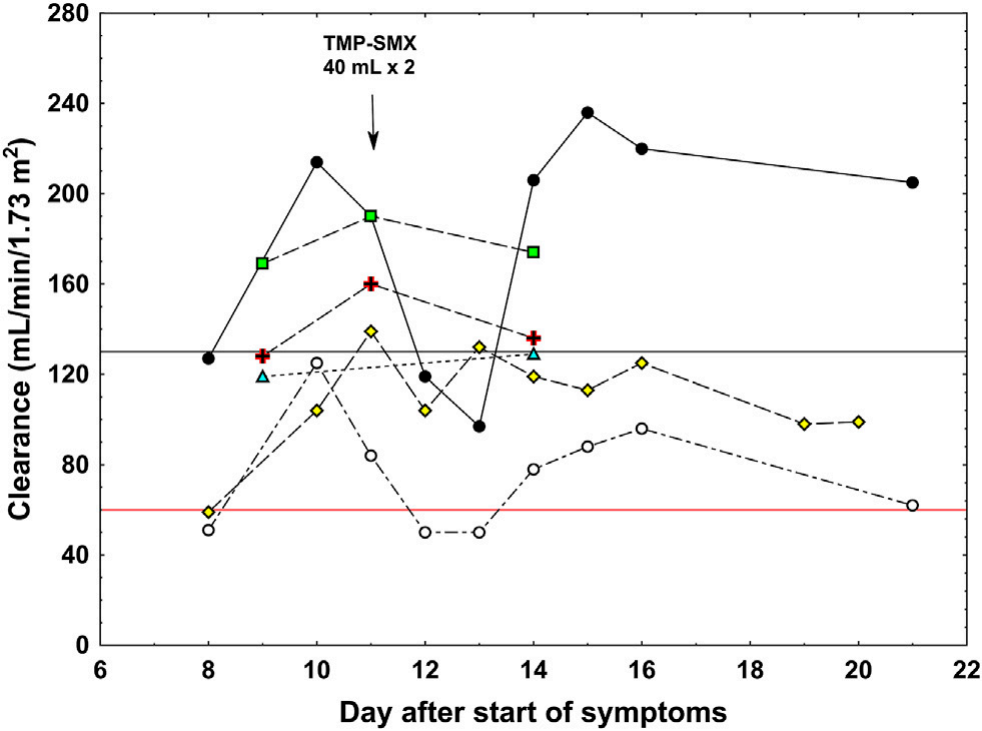
Clearance values in a patient with augmented renal clearance. Black circles: measured creatinine clearance, white circles: measured urea clearance, green triangles: iohexol clearance, yellow rhomboids: cystatin C clearance, red cross: total vancomycin clearance, green squares: free vancomycin clearance. The black horizontal line shows the limit for augmented renal clearance (130 ml/min/1.73 m^2^) and the red horizontal line shows the upper normal reference for urea clearance (60 ml/min/1.73 m^2^). Arrow shows the dosage of trimethoprim–sulfamethoxazole (TMP-SMX).

Our patient participated in a prospective observational study on the pharmacokinetics of vancomycin in intensive care patients. Vancomycin clearance was determined over a period of 5 days on three occasions, and iohexol clearance on two (Figure 2). The period included the day when TMP–SMX was administered. ARC was diagnosed since creatinine clearance was above 130 ml/min/1.73 m^2^ apart from the days immediately following TMP–SMX treatment. Urea clearance was initially 86% above the upper reference limit (Koch et al., 1980) and followed the same pattern as creatinine clearance (Figure 2). However, iohexol and cystatin C clearances were normal.

Vancomycin clearance calculated from total plasma concentrations was higher than that of iohexol, cystatin C, and creatinine clearances after trimethoprim treatment (Figure 2). The clearance of free vancomycin was even higher, approaching that of creatinine during ARC, indicating that vancomycin underwent both glomerular filtration and tubular secretion. Unfortunately, the total and free vancomycin clearances were not determined each day after TMP–SMX administration, precluding the conclusion regarding the effect of trimethoprim on the systems responsible for the tubular transport of vancomycin.

## Discussion

### Clinical Observations

The incidence of brain abscess is 0.3–1.3 per 100,000 people per year, 70% being male with an average age of 34 years (Brouwer et al., 2014a). The pathophysiology of brain abscess varies, with pathogens invading the CNS directly or via the bloodstream. The spread from dental infection is usually direct, causing abscesses in the frontal area. Symptoms are often non-specific, but headache is prominent, and only 50% have fever. Altered consciousness and signs of elevated intracranial pressure may occur. Brain abscesses of dental origin are often caused by mixed infections, for example, anaerobic Gram-positive cocci, and aerobic staphylococci and streptococci (Brouwer et al., 2014b). In this case, *S. intermedius*, a common commensal in the oral cavity, was detected in samples from the abscess material, as in a recent study reporting the predominance of this agent in brain abscesses (Darlow et al., 2020). The broad-spectrum β-lactam antibiotic meropenem, with good CNS penetration, was given, but despite this, endogenous endophthalmitis was diagnosed a week after admission. A recent case report described how *S. constellatus* of dental origin caused endophthalmitis and brain abscesses (Chheda et al., 2011), and endogenous endophthalmitis caused by *S. intermedius* has been described after routine dental practice (Mali et al., 2015).

The trough values of vancomycin and meropenem were initially far below their suggested therapeutic ranges, although the patient received recommended doses (Table 2), and clearances of creatinine, urea, and free vancomycin were high (Figure 2). The patient’s BMI was 30.9 kg/m^2^ (height 197 cm, weight 120 kg), with a body surface area of 2.55 m^2^, but his serum creatinine the first four days after admission was remarkably low [49, 44, 42, and 41 µmol/L (0.55, 0.49, 0.48, and 0.46 mg/dl)]. Despite his serious infection, sarcopenia was judged not responsible as he was not bedridden and sat in a chair during daytime. On treatment with trimethoprim, a well-known inhibitor of tubular creatinine transport (Berglund et al., 1975), creatinine clearance normalized. Apart from the days immediately following TMP–SMX treatment, creatinine clearance was around 70% higher than iohexol and cystatin C, indicating substantial tubular excretion of creatinine (Figure 2). Total vancomycin clearance was around 45% higher than iohexol and cystatin C clearances, and clearance of free vancomycin was even higher at around 90% of that of creatinine. The high clearance of vancomycin and demand for high doses of both vancomycin and meropenem clearly indicated high tubular secretion of these two drugs. Lonsdale et al. (2013) reported a similar case. This case and our case illustrate the phenomenon of contemporary rapid elimination of vancomycin and meropenem in neurosurgical patients with ARC (Lin Wu et al., 2015).

### Tubular Cell Transport of Creatinine, Urea, Vancomycin, and Meropenem

The literature regarding the tubular transport of creatinine is extensive (Wang and Kestenbaum, 2018). Creatinine is largely cleared by glomerular filtration, and normally around 10% is actively excreted by tubular transport. In the steady state, the excretion matches muscle production. In humans, creatinine is transported across the basolateral membrane of the proximal tubule by the human organic cation transporter-2 (hOCT2) (Urakami et al., 2004; Motohashi and Inui, 2013). The antiport transporters MATE1 and MATE2-K regulate its transport across the apical membrane into the tubular lumen. Trimethoprim is a known inhibitor of this transport system; hence, the temporary reduction in creatinine clearance to normal after trimethoprim treatment clearly shows that tubular creatinine transport was upregulated in this case (Figure 2).

Urea is also freely filtered in the glomeruli, but the concentrations along the tubular fluid transport system vary greatly due to resorption of water and removal of urea by transporters in the inner medullary collecting duct cells. The urea transporter UT-A1, at the apical membrane, transports urea from the lumen into the cell, while UT-A3 transports urea across the basal membrane to the interstitium. The final elimination of urea in the urine is around 30–50% of the glomerular filtrate in mammals (Klein et al., 2011). Surprisingly, urea clearance was also affected by treatment with trimethoprim in a similar way as creatinine clearance (Figure 2). The significance of this is unclear and, to our knowledge, has not been reported previously.

Drug transport within the tubular cells is the first fundamental stage in the onset of the nephrotoxic process. Knowledge of these concepts is important for the prevention of iatrogenic kidney damage, particularly in patients with underlying disease receiving concomitant treatment with several potentially nephrotoxic drugs (Fanos and Cataldi, 2001). In experimental models of nephrotoxicity, vancomycin crosses the basolateral membrane of the proximal tubular epithelium via the OCT system (Sokol, 1991). The involvement of MATE1 and MATE2-K proteins in the secretion of vancomycin has yet to be demonstrated (Pais et al., 2020). In our patient, vancomycin and creatinine clearances were simultaneously increased, suggesting that these transporters are also involved; otherwise, intracellular accumulation would have led to toxicity.

In an *in vitro* study, Shibayama et al. (2007) showed that hOCT1 and hOCT2 transport meropenem but were not able to examine transport across the apical membrane into the lumen. Uchino et al. (2000) reported that Npt1, an inorganic phosphate transporter, participates in the renal secretion of penem antibiotics. While it is known that renal excretion of meropenem is largely through active transport, the mechanism behind the rapid elimination of meropenem in our patient with ARC remains unclear.

### On the Causes of and Factors Contributing to Augmented Renal Clearance

There is no consensus in the literature regarding the causes of ARC. Recently, Cook and Hatton–Kolpek (2019) addressed the risk factors and potential contributing factors to the emergence of ARC. Chen and Nicolau (2020) published a review on ARC and put forward suggestions on how to identify ARC. There is further no consensus regarding how to define ARC. It should be kept in mind that low–molecular weight endogenous compounds (like creatinine) and drugs (such as vancomycin and meropenem) can be eliminated both by glomerular filtration and tubular secretion. Nevertheless, there is currently a broad consensus in considering the measured creatinine clearance of 130 ml/min/1.73 m^2^ as the lower limit for the diagnosis of ARC. We therefore fully adopt the definition of ARC as creatinine clearance slightly above the upper normal reference limit for GFR, that is, >130 ml/min/1.73 m^2^, as suggested by the Australian team and further advocated by them (Silva et al., 2020) and others (Mahmoud and Shen, 2017; Bilbao-Meseguer et al., 2018; Chen and Nicolau, 2020). Thus, creatinine clearance above this limit indicates ARC, caused by either increased glomerular filtration or tubular secretion. Table 3 shows the clearance rates of our patient both in absolute (ml/min) and normalized (mL/min/1.73 m^2^) terms. The absolute clearance rates however may just signify high clearance rates due to the big size of the patient and does not by itself indicate ARC. It is therefore confusing that some authors equalize high clearance rates in absolute terms with ARC. Neither can demand for high dosage of drugs by itself define ARC. However, the absolute clearance of each patient is the basis for dosage of the drugs.

**TABLE 3:** Creatinine, urea, iohexol, cystatin C, and vancomycin (total and free) clearances together with plasma concentrations of creatinine, urea, and cystatin C and excretion of creatinine and urea during the 6 days of clearance studies.

Vancomycin study day	1	2	3	4	5	6
Day from start of symptoms	9	10	11	12	13	14
Plasma creatinine (µmol/L)	46	44	44	59	51	43
Creatinine clearance (ml/min)	-	305	272	170	139	294
Creatinine clearance (mL/min/1.73 m^2^)	-	214	190	119	97	206
Urine excretion of creatinine, mmol/24 h		19.2	17.2	14.2	10.2	18.2
Plasma urea (mmol/L)		2.1	2.6	3.5	2.5	2.4
Urea clearance (ml/min)		179	119	71	71	111
Urea clearance (mL/min/1.73 m^2^)		125	84	50	50	78
Urine excretion of urea, mmol/24 h		536	447	360	256	384
Iohexol clearance, mL/min	177^a^	-	-	-	-	189
Iohexol clearance (mL/min/1.73 m^2^)	119^a^	-	-	-	-	129
Plasma cystatin C (mg/L)	-	0.79	0.61	0.79	0.64	0.7
Cystatin C clearance (ml/min)	-	154	206	153	195	174
Cystatin C clearance (mL/min/1.73 m^2^)	-	104	139	104	132	119
Vancomycin clearance (ml/min)	190	-	237	-	-	199
Vancomycin clearance (mL/min/1.73 m^2^)	128	-	160	-	-	136
Free vancomycin clearance (ml/min)	251	-	281	-	-	254
Free vancomycin clearance (mL/min/1.73 m^2^)	169	-	190	-	-	174

a Calculated from iohexol given at the Department of Radiology.

One suggested and cherished cause of ARC is an increased cardiac output that would give increased renal blood flow, and thus increased GFR (Sime et al., 2015; Atkinson, 2018). In our patient, an echocardiogram was performed that showed no signs of endocarditis. At this investigation, the cardiac output was considered normal with normal contractility, and there were no signs of organic heart failure. We have measured GFR with the best available method (iohexol plasma clearance), and thus, we exclude the increased cardiac output with increased GFR as the cause of ARC in our patient. The GFR measured by iohexol clearance was quite normal, and the creatinine clearance after trimethoprim treatment decreased to half its value into the area of normal GFR. A similar result was found with urea clearance. We therefore consider the cause of ARC in our patient to be increased tubular secretion. The next question however is whether this is constitutional in our patient or induced during the current period of illness. We reviewed the earlier medical records of our patient and found seven serum creatinine values with a mean ± SD of 68.1 ± 13.0 µmol/L during the years 2012–2019. The mean serum creatinine of 44.2 ± 5.7 µmol/L during the present hospital stay was significantly lower than that during the earlier period (*p* < 0.001). Furthermore, after a follow-up of 16 months, his plasma creatinine was 69 µmol/L, creatinine clearance was 110 ml/min/1.73 m^2^, urea clearance was 48 ml/min/1.73 m^2^, and iohexol clearance was 88 ml/min/1.73 m^2^, that is, quite normal results. It therefore seems that during the stay at neurosurgical unit, he had a period of induced increased tubular secretion of creatinine as the dominant cause of ARC. The conclusion of increased tubular secretions in our patient however does not preclude that increased GFR occurs in other patients. In a recent publication, it was shown that 29 patients had ARC as measured by 6-hour creatinine clearance. Of these, 16 patients had hyperfiltration and 13 had not, as measured by iohexol clearance (Collet et al., 2021). Thus, ARC can be due to increased GFR or increased tubular secretion or both. Our study indicates that this may also be true for drugs such as vancomycin and meropenem. To discriminate between these possibilities, employment of a highly standard GFR method is necessary, as well as actual measurement of creatinine or drug clearances that can indicate augmented clearance beyond the patients true GFR.

## Conclusion

This case report describes a 34-year-old man with multiple brain abscesses where the clearances of creatinine, urea, and vancomycin were all increased. High demand for meropenem to reach optimal plasma concentration also indicated increased elimination rate. The increased elimination of these compounds was not due to increased GFR as shown by concomitant measured normal iohexol and cystatin C clearances. We therefore infer that the increased clearances were due to increased tubular secretion. For creatinine, this is proven by the fact that during the study period, temporarily given trimethoprim lowered the clearance of creatinine to normal. High clearance of urea that fell into normal was also found after trimethoprim treatment. However, tubular transport and secretion of urea into the urine are quite different from those of creatinine, and there is no information available in the literature on trimethoprim effects on tubular transporters of urea. This is a new finding and needs to be corroborated in larger studies. Anyhow, urea clearance was higher than normal, and this by itself infers increased excretion into the urine by the tubular system. To our knowledge, there is no reliable information available on human tubular transporters of vancomycin. We have no data on vancomycin clearance the immediate days after trimethoprim dosing, so we cannot evaluate the possible effect of trimethoprim on tubular transport of this drug. However, vancomycin clearance as calculated from non–protein-bound vancomycin was high, above normal GFR and 130 ml/min/1.73 m^2^, set for definition of ARC, and close to the level of creatinine clearance. Thus, our study shows increased tubular secretion of vancomycin, but we cannot indicate the mechanism for its tubular secretion. A limitation of our study is that the clearance rates of meropenem were not actually measured.

We conclude that tubular secretion is responsible for the clearance rates beyond the true normal GFR. Further studies on tubular secretion are needed to understand the mechanisms for increased tubular secretion of drugs in intensive care patients with ARC. Increased clearances of drugs like vancomycin and β-lactam antibiotics may result in life-threatening subtherapeutic antibiotic concentrations. Finally, dose recommendations for renally excreted drugs in ARC patients should be implemented to prevent treatment failure, increased morbidity, and mortality.

## Data Availability

The original contributions presented in the study are included in the article/supplementary material; further inquiries can be directed to the corresponding author.

## Abbreviations

ARC, augmented renal clearance; BSA, body surface area; CNS, central nervous system; CRP, C-reactive protein; CSF, cerebrospinal fluid; OCT, organic cationic transporter; hOCT1, human organic cation transporter-1; hOCT2, human organic cation transporter-2; MATE1, multidrug and toxin extrusion 1; MATE2-K, multidrug and toxin extrusion 2-K; MRI, magnetic resonance imaging; HIV, human immunodeficiency virus; TMP-SMX, trimethoprim–sulfamethoxazole; UT-A1, urea transporter A1; UT-A3, urea transporter A3.
